# Tumor necrosis factor-α enhances the expression of vascular endothelial growth factor in a mouse orthodontic tooth movement model

**DOI:** 10.1016/j.jds.2021.08.011

**Published:** 2021-09-01

**Authors:** Takahiro Noguchi, Hideki Kitaura, Aseel Marahleh, Fumitoshi Ohori, Yasuhiko Nara, Adya Pramusita, Ria Kinjo, Jinghan Ma, Kayoko Kanou, Itaru Mizoguchi

**Affiliations:** Division of Orthodontics and Dentofacial Orthopedics, Department of Translational Medicine, Tohoku University Graduate School of Dentistry, Sendai, Japan

**Keywords:** Tooth movement, Orthodontics, Mouse, VEGF, Osteoclast, Bone resorption

## Abstract

**Background/purpose:**

Tooth movement that is achieved using orthodontic mechanical principles relies on bone resorption which takes place on the compression side via osteoclasts. Tumor necrosis factor-α (TNF-α) has been known to affect osteoclast formation in orthodontic tooth movement (OTM). Vascular endothelial growth factor (VEGF), which is one of the mediators of angiogenesis, also plays an important role in OTM by inducing vascular permeability and chemotaxis of osteoclast precursors. Therefore, the purpose of this research was to evaluate the effect of TNF-α on VEGF expression during OTM.

**Materials and methods:**

In order to demonstrate the effect of TNF-α on VEGF expression during OTM, a nickel titanium closed coil spring was fixed to the upper left first molar and the alveolar bone beneath the upper incisors of both wild type (WT) and TNF receptors (TNFRs) deficient mice resulting in a mesial movement of the molar for 12 days. The maxilla was removed for histological analysis and real-time RCR analysis of VEGF expression.

**Results:**

Immunohistochemical analysis revealed that there were fewer VEGF-positive cells in the periodontal membrane on the mesial side of the distobuccal root in TNFRs-deficient mice than that in WT mice during the OTM for 12 days. Furthermore, expression of VEGF mRNA is lower level in TNFRs-deficient mice than that in WT mice.

**Conclusion:**

Our results indicate that TNF-α plays an important role in VEGF expression during tooth movement.

## Introduction

Orthodontic tooth movement (OTM) occurs with bone resorption and bone apposition of the alveolar bone and periodontal ligament when orthodontic forces are applied. Orthodontic forces activate numerous molecules such as growth factors, cytokines, neurotransmitters, and bone matrix components. The function and differentiation of osteoclasts and osteoblasts which lead to bone resorption and bone forming were involved by some of these molecules.^1^, ^2^, ^3^ The osteoclast, a multinucleated derivative of the hematopoietic stem cells, is a prerequisite for bone remodeling through resorption of the bone matrix. Differentiation and functional maturation of osteoclasts depend on two principal cytokines; macrophage colony stimulating factor (M-CSF) and receptor activator of nuclear factor-kappa B ligand (RANKL) which without osteoclastogenesis doesn't occur.^4^ Tumor necrosis factor-α (TNF-α) has also been reported to promote differentiation of osteoclasts from osteoclast precursors derived from bone marrow cells *in vitro.*^5^, ^6^, ^7^ TNF-α-induced osteoclast formation can be central to osteoerosive disorders such as rheumatoid arthritis, postmenopausal osteoporosis, periprosthetic bone loss, and periodontal disease.^8^, ^9^, ^10^, ^11^

Vascular endothelial growth factor (VEGF) possesses angiogenic and vasculogenic functions under both physiological and pathological conditions.^12^, ^13^, ^14^ VEGF concentrations are higher in the serum and synovial fluid of rheumatoid arthritis patients than in that of osteoarthritis patients or healthy persons.^15^, ^16^, ^17^, ^18^ The pro-inflammatory cytokine TNF-α is also elevated in rheumatoid arthritis patients.^19^ TNF-α has been reported to induce the expression of VEGF in the synovial fluid of patients with rheumatoid arthritis. Furthermore, VEGF induces TNF-α expression in the synovial fluid of these patients.^20^ Thus, TNF-α and VEGF are important factors in the progression of rheumatoid arthritis.

Several studies have shown that orthodontic force induces TNF-α expression, suggesting an important role for TNF-α in OTM.^21^, ^22^, ^23^ A mouse OTM model was established to investigate the mechanism of bone remodeling during OTM.^24^^,^^25^ This leads us to investigate the role of TNF-α through developing an OTM mouse model which lacks both TNF-α receptors termed TNF receptors (TNFRs)-deficient mice.^24^^,^^25^ The results showed that OTM was decreased in TNFRs-deficient mice in comparison with wild-type (WT) mice. These reports concluded that TNF-α plays a crucial role in OTM-induced osteoclast formation and bone resorption.^24^, ^25^, ^26^, ^27^

In this study, we evaluated that the expression of VEGF in the mouse OTM model using WT mice and TNFRs-deficient mice.

## Materials and methods

### Mice

WT male C57BL6/J mice were obtained from CLEA Japan (Tokyo, Japan) and TNFRs deficient mice (Tnfrsf1a^*tm1lmx*^Tnfrsf1b^*tm1lmx*^) were purchased from the Jackson Laboratory (Bar Harbor, ME, USA). OTM were performed on 8 to 10-week-old mice. The mice were fed a granular diet (Oriental Yeast, Tokyo, Japan) to prevent eating difficulties in OTM. All experimental procedures were performed in accordance with Tohoku University Regulation.

### Orthodontic tooth movement in mouse

**Figure 1:**
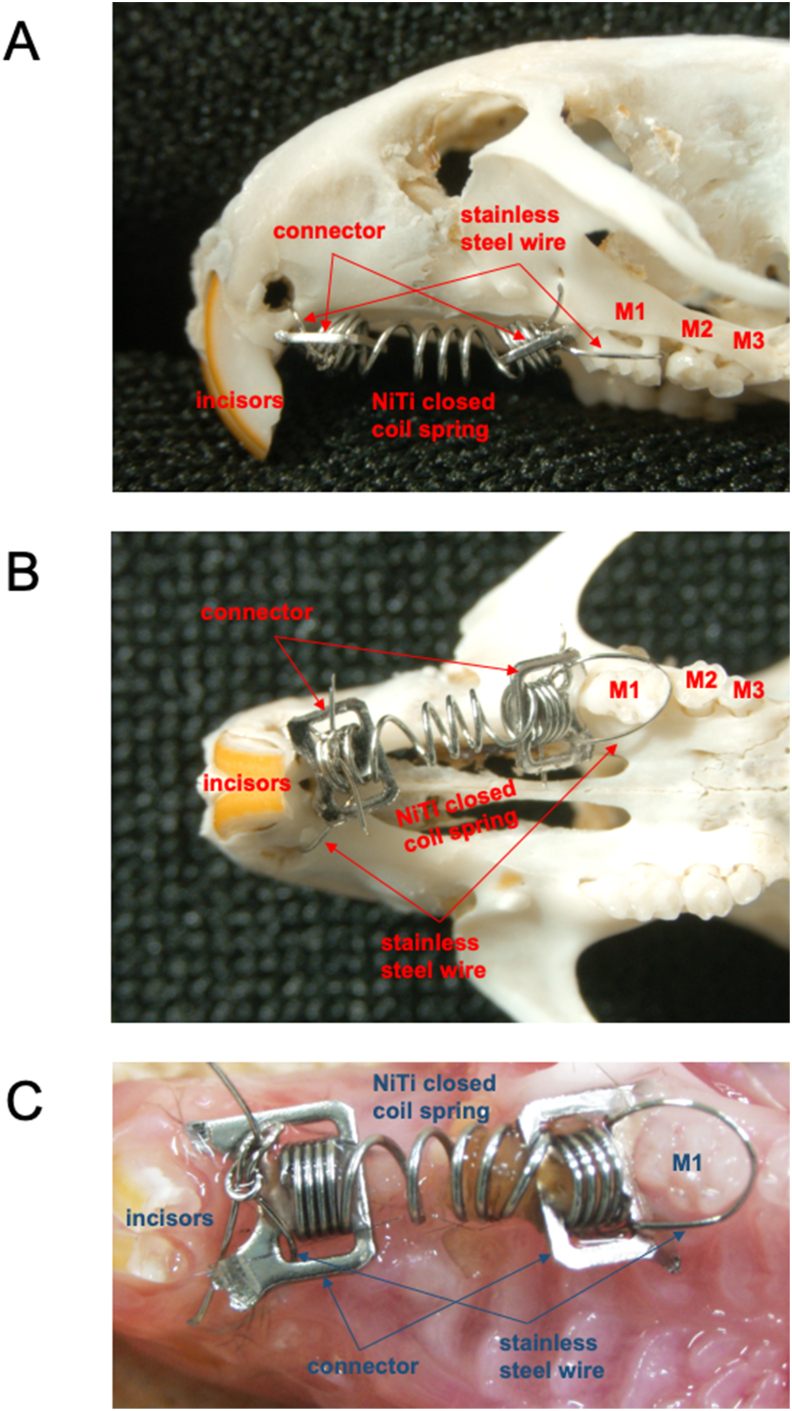
Photograph of appliance for orthodontic tooth movement in mice. A closed Ni–Ti coil spring is attached between the upper left first molar and holes in the anterior alveolar bone by tying 0.1 mm ligature wires. (A) Buccal view of the dry skull model with the appliance attached. (B) Occlusal view of the dry skull model with the appliance attached. (C) Intra-oral photograph of the appliance. M1, first molar; M2, second molar; M3, third molar.

Mice were anesthetized with 4.0 mg/kg of midazolam, 0.3 mg/kg of medetomidine, and 5.0 mg/kg of butorphanol. Mice were put a nickel-titanium (Ni–Ti) closed coil spring (Tomy, Fukushima, Japan) was fixed to the left maxillary first molar, and the maxillary alveolar bone underneath the incisors for the application of OTM under anesthesia. For appliance fixation, a holes were drilled in alveolar bone on palatal side of the two incisors with a 0.8 mm round steel bur. The connectors attached to the coil spring was tied to a 0.1 mm stainless steel wire through these holes (Fig. 1).^26^, ^27^, ^28^, ^29^, ^30^ According to manufacturer's instructions, the Ni–Ti closed coil spring has a 10 g force. OTM was carried out for 12 days. The experiment design contained four groups and each group had four mice.

### Preparation for histological observation

The mice were euthanized by an overdose of 5% isoflurane in an inhalation chamber. Using scissors and forceps, the skin of mouse at the head was grasped and cut the neck. Using scissors and forceps, the mandible and maxilla was cut off from the skull. The maxillae were put in not less than 10 mL of paraformaldehyde (PFA) 4% (diluted in PBS) for 3 days for fixation. Maxillae were decalcified in 14% ethylenediaminetetraacetic acid (EDTA) 0.5 M pH 8.0 for 1 month at room temperature. The EDTA solution was changed every 2 days. Decalcified maxillae were put in histological cassettes and place them in a bag. Immerse the samples in 1000 mL of 30% ethanol, 70% ethanol, 80% ethanol, and 90% ethanol for 1 h each, 1000 mL of 95% ethanol for 3 h, 1000 mL of 100% ethanol twice for 7 h and 12 h each, 1000 mL of xylene three times for 0.5 h, 1 h and 1.5 h each for dehydration, and then 1000 mL of liquid paraffin (56 °C) twice for 7 h and 12 h each in an automatic tissue processor connected to a chemical hood to allow the xylene to evaporate. The samples were embedded in paraffin using a histological blocking machine. The samples were started cutting 20 μm thick horizontal sections through the maxilla until the area of the bifurcation of the distobuccal root is revealed. At this point decrease the cutting thickness to 4 μm and proceed sectioning all the way to the apex of the root.^27^^,^^29^ The sections were deparaffinized and hydrated through xylenes and decreasing concentrations of graded ethanol series. For VEGF staining, the sections were immersed in 0.3% H_2_O_2_ in PBS for 15 min and were blocked with blocking solution (5% skim milk, 0.05% Triton X-100, 3% bovine serum albumin in PBS) for 30 min at 37 °C. After blocking, the sections were incubated with rabbit monoclonal anti-VEGFA antibody (ab232858 Abcam, Cambridge, MA, USA) 1:500 diluted in blocking solution at 4 °C overnight for immunohistochemistry. After washing, the sections were processed with VECTASTAIN Elite ABC Kit PK 6101 (Vector, Burlingame, CA, USA). The slides were treated with 3,3′-diaminobenzidine substrate (Nichirei Biosciences, Tokyo, Japan) and watched for staining, and then rinse slides in distilled water. The sections were counterstained with hematoxylin for 10 s and rinse in distilled water. The number of VEGF-positive cells at the periodontal membrane on the compression side of the distobuccal root on day 12 during OTM was evaluated.

### Total RNA preparation and real-time RT-PCR analysis

Alveolar bones including soft tissue around the maxillary left first molar were extracted at 12 days after OTM to evaluate VEGF expression around the maxillary left first molar. For extraction of total RNA, alveolar bone including soft tissue around the maxillary left first molar was dissected and frozen in liquid nitrogen after mice were sacrificed. The frozen alveolar bones including soft tissue were crushed and homogenized by a Micro Smash MS-100R (Tomy Seiko Co. Ltd., Tokyo, Japan). These samples were suspended in TRIzol Reagent (Invitrogen, Carlsbad, CA, USA). Total RNA was isolated and purified from samples using RNeasy Mini Kit (Qiagen, Valencia, CA, USA) as described previously.^29^ cDNA synthesized from 2 μg of total RNA was used for cDNA synthesizing by SuperScript IV Reverse Transcriptase (Invitrogen). TB Green Premix Ex Taq II (Takara, Shiga, Japan) with the Thermal Cycler Dice Real Time System (Takara) were used for real-time PCR. PCR was performed as follows: initial denaturation stage (30 s at 95 °C), amplification stage (50 amplification cycles with each cycle composed of a denaturation step of 5 s at 95 °C and an annealing step of 30 s at 60 °C) and final dissociation stage (15 s at 95 °C, 30 s at 60 °C and 15 s at 95 °C). Glyceraldehyde 3-phosphate dehydrogenase (GAPDH) mRNA was used for normalizing expression levels. The following primers were GAPDH, 5′-GGTGGAGCCAAAAGGGTCA-3′ and 5′-GGGGGCTAAGCAGTTGGT-3′;^26^ VEGF, 5′- GTGCACTGGACCCTGGCTTTA-3′ and 5′-GGTCTCAATCGGACGGCAGTA-3′.^31^

### Statistical analysis

The Scheffe's F test was performed to evaluate significance. Statistical significance was assumed at a threshold of p < 0.05. All quantitative results are presented as the mean ± standard deviation.

## Results

### TNF-α enhances VEGF expression during OTM

**Figure 2:**
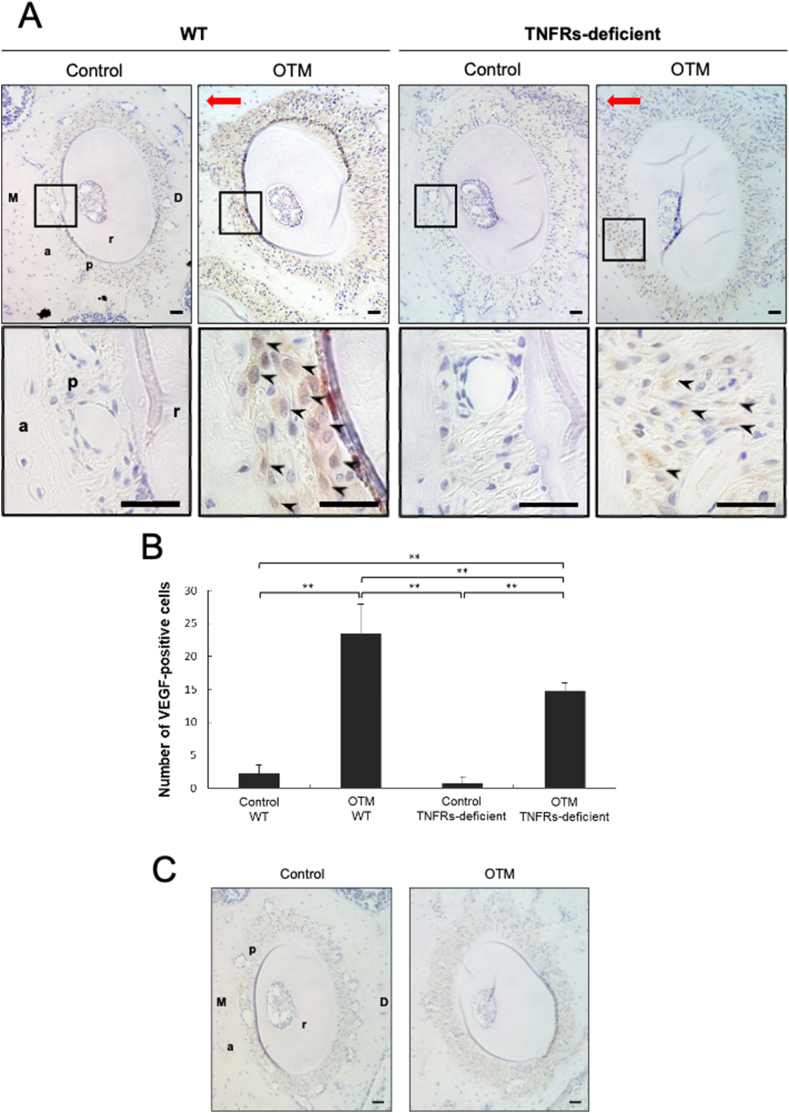
Immunohistochemical image of the distobuccal root of the upper left first molar of tumor necrosis factor-α receptors (TNFRs)-deficient mice after orthodontic tooth movement (OTM) for 12 days compared with that of wild-type (WT) mice. (A) Represents sections of the distobuccal root stained with anti-vascular endothelial growth factor (VEGF) antibody and counterstained with hematoxylin. (B) The number of VEGF-positive cells located on the alveolar bone surface after OTM for 12 days in WT and TNFRs-deficient mice. (C) Immunohistochemical image of the distobuccal root of the upper right molar of WT mice (with drilling) after OTM for 12 days compared with that of WT control mice (without drilling). Represents sections of the distobuccal root stained with anti-VEGF antibody and counterstained with hematoxylin. Black arrow heads = VEGF-positive cells; red arrows = direction of orthodontic force; M, mesial side; D, distal side; a, alveolar bone; p, periodontal ligament; r, root. Scale bars = 20 μm. The data are expressed as the mean ± standard deviation. Statistical significance was determined by Scheffe's test (n = 4, ∗∗P < 0.01).

**Figure 3:**
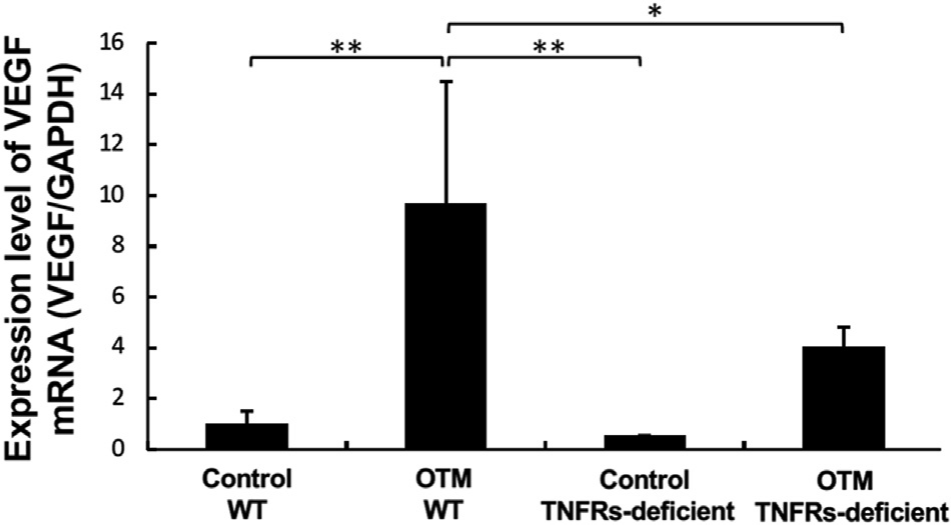
Effect of OTM on expression of VEGF mRNA. Relative expression of VEGF mRNA in alveolar bones including soft tissue around the maxillary left first molar with OTM for 12 days or without OTM in WT and TNFRs-deficient mice by real-time PCR. VEGF mRNA levels were normalized to the levels of GAPDH. Statistical significance was determined by Scheffe's test (n = 4, ∗∗P < 0.01).

The OTM mouse model uses a nickel-titanium (Ni–Ti) closed-coil spring which is fixed to the upper anterior alveolar bone and the upper left first molar to move the molar mesially. WT and TNFRs-deficient mice are compared to evaluate the role of TNF-α in VEGF expression during OTM. Immunohistochemical analysis showed that there were significantly fewer VEGF-positive cells in the periodontal membrane on the mesial side of the distobuccal root in TNFRs-deficient mice than that in WT mice during 12 days of OTM (Fig. 2A and B). In this method, holes are drilled through the alveolar bone palatally to the upper incisors. There is a possibility that the periodontal membrane of the first molars was influenced by inflammation caused by drilling holes. Therefore, it was checked VEGF expression at mesial side of periodontal membrane of first molar in opposite side (right side) of tooth movement mice and control mice for evaluation of effect of inflammation caused by drilling. There is no difference of VEGF expression between opposite side of OTM mice and control mice (Fig. 2C). On day 12 of OTM, expression of VEGF mRNA was greater in WT mice when compared with that of TNFRs-deficient mice. The expression of VEGF mRNA in WT mice and TNFRs-deficient mice on day 12 of OTM was greater than that observed for WT mice and TNFRs-deficient mice controls (0 day) (Fig. 3).

## Discussion

A mouse OTM model was established to investigate the mechanism of orthodontic force-induced bone remodeling.^24^ Previous reports described the role of TNF-α in OTM in TNFRs-deficient mice using the following tooth movement model.^24^^,^^25^ A Ni–Ti closed-coil spring was inserted between the upper incisors and the upper left first molar, and fixed with 0.1 mm stainless wire around the incisors and the molar. To prevent detachment of the appliance, 0.1 mm stainless wire was hooked to a shallow groove that was made approximately 0.5 mm above the gingiva and then fixed with dental adhesive. However, due to the growth of the incisors during tooth movement, the appliance often became detached from the incisors. A shallow groove was made again and the wire was rehooked to the new groove every 4 days during the experiment. This involved significant effort, and the incisors were sometimes broken. Therefore, the method was improved by attaching the appliance between the alveolar bone near the incisors and the upper left first molar with a 0.1 mm stainless wire to avoid rehooking the wire.^26^, ^27^, ^28^, ^29^, ^30^ This saves effort and prevents breakage of the incisors. Therefore, we improved the method. In the present method, holes are drilled through the alveolar bone palatally to the upper incisors. In this drilling area, there is a nasal-associated lymphoid tissue. There is a possibility to influence the periodontal membrane of the first molars by inflammation caused by drilling holes at the each of incisors at the alveolar bone. These results showed that there is no difference of expression of VEGF between opposite side of OTM mice and control mice. Therefore, it was suggested that there is less effect of inflammation caused by drilling each of the two incisors at the alveolar bone for periodontal membrane of first molars.

Various reports have shown that TNF-α is induced by orthodontic force in OTM, suggesting that TNF-α plays a crucial role in OTM.^22^^,^^23^^,^^32^, ^33^, ^34^, ^35^ It has been reported that TNF-α plays a notable role in orthodontic force-loaded teeth in an OTM mouse model in which TNFRs-deficient mice exhibited less tooth movement than WT mice.^24^, ^25^, ^26^, ^27^ One of these studies used immunohistochemistry to show that TNF-α is expressed in the periodontal membrane during OTM.^24^ Several studies have shown that TNF-α induces VEGF. It has been reported that TNF-α induces VEGF gene expression in human glial cells.^36^ It has also been reported that TNF-α induces expression of VEGF, and that VEGF induces TNF-α expression, in the synovial fluid of rheumatoid arthritis patients.^20^ In this study, a significantly smaller number of VEGF-positive cells were found on the mesial side of the distobuccal root in TNFRs-deficient mice as compared to WT mice after OTM for 12 days as analyzed via immunohistochemical staining. Thus, orthodontic force-induced VEGF expression was inhibited in TNFRs-deficient mice. These results suggest that TNF-α may induce VEGF expression during OTM.

It has also been reported that VEGF induces RANKL expression in synovial fibroblasts, suggesting that VEGF indirectly induces osteoclast formation in rheumatoid arthritis synovium.^37^ The report also showed that VEGF directly enhanced osteoclast formation from peripheral blood monocytes.^37^ In the present study, VEGF expression was induced during tooth movement, suggesting that orthodontic force-induced VEGF might induce osteoclast formation, thus enhancing bone resorption and tooth movement.

In conclusion, TNF-α was shown to play a significant role in VEGF expression in OTM. VEGF may enhance osteoclast formation, bone resorption, and tooth movement during osteoclast formation.

## Declaration of competing interest

The authors declare that this research was conducted in the absence of any relationships that could be construed as a potential conflict of interest.
